# Transplacental Gene Delivery (TPGD) as a Noninvasive Tool for Fetal Gene Manipulation in Mice

**DOI:** 10.3390/ijms20235926

**Published:** 2019-11-25

**Authors:** Shingo Nakamura, Satoshi Watanabe, Naoko Ando, Masayuki Ishihara, Masahiro Sato

**Affiliations:** 1 Division of Biomedical Engineering, National Defense Medical College Research Institute, Saitama 359-8513, Japan; naoandokoro@gmail.com (N.A.); ishihara@ndmc.ac.jp (M.I.); 2 Animal Genome Unit, Institute of Livestock and Grassland Science, National Agriculture and Food Research Organization (NARO), Tsukuba, Ibaraki 305-0901, Japan; kettle@affrc.go.jp; 3 Section of Gene Expression Regulation, Frontier Science Research Center, Kagoshima University, Kagoshima 890-8544, Japan; masasato@m.kufm.kagoshima-u.ac.jp

**Keywords:** CRISPR/Cas9, fetal gene therapy, genome editing, transplacental gene delivery (TPGD), TPGD for acquiring genome-edited fetuses (TPGD-GEF)

## Abstract

Transplacental gene delivery (TPGD) is a technique for delivering nucleic acids to fetal tissues via tail-vein injections in pregnant mice. After transplacental transport, administered nucleic acids enter fetal circulation and are distributed among fetal tissues. TPGD was established in 1995 by Tsukamoto et al., and its mechanisms, and potential applications have been further characterized since. Recently, discoveries of sequence specific nucleases, such as zinc-finger nuclease (ZFN), transcription activator-like effector nucleases (TALEN), and clustered regularly interspaced short palindromic repeats (CRISPR)/CRISPR-associated protein-9 nuclease (Cas9) (CRISPR/Cas9), have revolutionized genome editing. In 2019, we demonstrated that intravenous injection of plasmid DNA containing CRISPR/Cas9 produced indels in fetal myocardial cells, which are comparatively amenable to transfection with exogenous DNA. In the future, this unique technique will allow manipulation of fetal cell functions in basic studies of fetal gene therapy. In this review, we describe developments of TPGD and discuss their applications to the manipulation of fetal cells.

## 1. Introduction

Genome editing enzymes such as zinc-finger nuclease (ZFN), transcription activator-like effector nuclease (TALEN), and clustered regularly interspaced short palindromic repeats (CRISPR)/CRISPR-associated protein-9 nuclease (Cas9) (CRISPR/Cas9), have been successfully used to manipulate genomes with unprecedented precision [1]. In the field of human gene therapy, the feasibility of genome editing in primary human hematopoietic cells is of great interest due to the potential to treat human genetic disorders [2]. In previous studies of these technologies, applications to the prevention of human immunodeficiency virus (HIV)-1 infection in hematopoietic cells were investigated after transfection with ZFN [3,4]. Moreover, targeted genome editing for the treatment of acute lymphoblastic leukemia was achieved using TALEN [5]. Among genome editing tools, CRISPR/Cas9 is considered appropriate for genome editing in vivo and in vitro, because the design of guide (g)RNAs and construction of nuclease/gRNA complexes is easier than for ZFN, and TALEN [6]. This technology has also been considered as a promising tool for human gene therapy [7].

In utero gene therapy offers several advantages in the treatment of genetic disorders, because large numbers of somatic stem cells are readily available for gene transfer in the fetus. Moreover, permanent replacement of genes in somatic stem cells will ensure that daughter cells carry the gene, obviating the need for repeated therapy in affected individuals [8]. In addition, fetuses may be especially amenable to gene therapy because the immunological hematopoietic system is immature during gestation, precluding immune reactions toward the transgene [9]. 

The development of efficient methods for the transfer of nucleic acids to fetuses is an important goal for in utero gene therapy [10]. A number of animal models have been developed to evaluate new gene delivery methods, which include direct injections of exogenous DNA into fetuses [11,12,13], injections of DNA into the placenta or umbilical cord [14,15], and injections of DNA into the amniotic cavity [16,17] or the yolk sac [17]. All of these approaches require ex vivo handling procedures that temporally expose fetuses, and micropipette injections of nucleic acids under anesthetic conditions are time-consuming and labor-intensive. As an alternative administration route, transplacental transfer of plasmid DNA constructs was reported after tail-vein injections into dams. This approach represents a noninvasive and convenient method for the transfection of fetal tissues [18]. After transplacental transport, administered nucleic acids reach the fetal circulation and efficiently transfect fetal cells. This novel approach is hereafter referred to as transplacental gene delivery (TPGD). Since the work of Tsukamoto et al. (1995) [18], supporting data have been generated, and TPGD has been achieved with nucleic acids derived from plasmids or viral vectors. Very recently, Nakamura et al. (2019) elicited CRISPR/Cas9-mediated mutations in a target locus of embryonic cells for the first time using TPGD for acquiring genome-edited fetuses (TPGD-GEF) [19]. This unique gene delivery technique may be a useful tool for manipulating embryonic cell functions in vivo, with simpler procedures for generating transgenic animals and potential for the treatment of fetal disease. In this review, we summarized developments of TPGD and discussed the possibility of fetal gene therapy using the CRISPR/Cas9 system.

## 2. TPGD

### 2.1. Past Achievements 

To our knowledge, there are currently only 13 published reports concerning TPGD (Table 1) [9,10,18,19,20,21,22,23,24,25,26,27,28]. In their seminal study of 1995, Tsukamoto et al. demonstrated that exogenous plasmid DNA containing a gene for lacZ (coding for β-galactosidase) can be complexed with liposome and transferred to fetuses via the placenta following single tail-vein injections into pregnant females [18]. Some of the fetuses in their study exhibited blue deposits throughout the body, indicating successful gene delivery and expression. Moreover, these procedures for gene transfer into post-implantation embryos are comparatively simple and allow rapid analyses of the effects of transgenes on fetuses. 

In subsequent reports, nucleic acids were successfully delivered to post-implantation embryos and fetuses. In particular, Okuda et al. (2001) examined fetal uptake of plasmid DNA in complexes with cationic liposomes until the 21st post-coital day but observed little transfer during early pregnancy [9]. Instead, considerable numbers of injected cationic liposomes were present in the tissues of fetuses at E9.5 (E0 of gestation is defined as the day on which the copulation plug is found). These observations were consistent with those of Tsukamoto et al. (1995) [18]. Analyses of immune responses of progeny whose mothers had been immunized with the influenza DNA vaccine indicated enhanced protection against the same viral infection. Kikuchi et al. (2002) performed TPGD using the transgenic (Tg) mouse line CETZ-17, which contains transgenes for a chicken β-actin promoter, the *loxP*-flanked enhanced green fluorescent protein (*eGFP*) cDNA/chloramphenicol acetyltransferase (*CAT*) gene, the *lacZ* gene, and poly (A) sites [21]. In this study, B6C3F1 hybrid female mice were mated with CETZ-17 males and transplacental transfer of a Cre-expressing plasmid DNA construct was achieved with FuGENE6 lipid reagent. Subsequently, lacZ expression was detected in some of the fetuses (~24%), especially in heart and circulatory tissues. These observations confirmed Cre/*loxP*-mediated excision of the CETZ-17 transgene by TPGD. Similarly, O’Shea et al. (2006) systemically administered short hairpin (sh)RNAs to mothers during the early post-implantation stage of gestation and observed gene knockdown and defects that resembled those in null embryos [10]. These investigators targeted the Sex-determining region Y (*Sry*) gene, which is responsible for the initiation of male sex determination in mammals. Knockdown of this gene resulted in feminization of gonad development in mouse embryos. The authors concluded that systemic delivery of shRNAs is a feasible approach for gene silencing in embryos. These experiments also suggested that TPGD could be used to achieve in vivo transfection of fetal gonadal cells (at least male cells). Currently, however, successful germ-line transmission of transferred genes has not been achieved using this approach.

### 2.2. Optimal Timing of TPGD 

Early studies of TPGD were designed to determine optimal stages at which fetuses are effectively transfected, and comparisons of early and late gestational stages have been reported. Initially, Tsukamoto et al. (1995) demonstrated high gene delivery efficiency of TPGD on E9.0 [18], when the fetal heart begins to function, and many other organs differentiate dramatically [29]. Fetuses treated at E9.0 contained at least 40 times more plasmid DNA than those treated on E12.0 or E15.0, and no plasmid DNA was detected in fetuses that were treated on E3.0 or E6.0 [18]. Moreover, gene transferred mice appeared normal from the time their dams were injected until at least 15 months after birth. In addition, the introduced plasmid DNA was undetectable in progeny (14 months after birth), as shown by Southern blot analyses. These results indicate transient expression of transgenes that are introduced using TPGD and no effects on fetal development. According to Kikuchi et al. (2002), lacZ was preferentially expressed due to Cre-mediated excision of *loxP*-flanked *eGFP*/*CAT* sequences in some fetal hearts. Moreover, circulatory tissues in vertebral areas were positively stained with the lacZ substrate X-Gal when TPGD was performed on E12.5 and E13.5 of pregnancy, although evidence for fetal gene delivery from dams was observed on E5.5 [21]. Notably, placental formation occurs at E12.5–E13.5 [29] and facilitates TPGD by increasing the accessibility of exogenous DNA to fetal vessels. Other groups performed TPGD at E9.5 (for delivery of RNAi) [25], at E12.5 (for delivery of adeno-associated virus (AAV) particles) [26], and at E11.5 (for delivery of plasmid DNA/liposome complexes) [20]. These studies suggest that TPGD fails to achieve ideal results at early stages of post-implantation development. Ideally, TPGD is recommended in mid-gestational stages, as in many cases, successful gene delivery to fetuses was achieved when TPGD was performed from E9.5 to E12.5.

### 2.3. Fetal Immune Responses by TPGD

DNA/lipid complexes have been delivered to fetuses via the placenta, leading to synthesis of plasmid encoded proteins in transfected fetal cells. Therefore, tail-vein injections of plasmid DNA during pregnancy may induce antigen-specific tolerance in their progeny. Mor et al. immunized pregnant mice by administering various plasmids at various doses and observed fetal immune responses (but not tolerance) against the DNA [30]. These observations suggest that DNA vaccines could prevent infections in children [31]. To address this hypothesis using TPGD, Okuda et al. (2001) administered plasmids encoding antigens from HIV-1 or influenza virus to pregnant mice (at E9.5) and determined whether antigen-specific acquired immunity was induced in fetuses [9]. Following immunization with DNA vaccine, progeny from vaccinated dams mounted stronger antigen-specific immune responses than those of non-vaccinated dams, leading to the development of resistance to influenza virus infection. Although liposome gene carriers may also act as adjuvants that enhance immune responses, these results suggest that DNA-vaccinated dams confer antigen-specific immunity to their progeny. Thus, exposure of pregnant dams to life-threatening pathogens or continuous perinatal expression of pharmacological proteins may be beneficial for fetal vaccination. In particular, this approach could be used to mitigate hepatitis B virus infections at the end of pregnancy, during birth, and during periods of breastfeeding. These infections contribute many deaths among first-week infants and become chronic in 90% of perinatally infected infants. Moreover, 25% of these infants die from related chronic liver disease as adults [32]. In experiments with pigs, Rinaldi et al. performed in utero gene delivery for DNA immunization using a pCMV-HBs plasmid, which expresses the hepatitis B surface antigen (HBs) under the control of the cytomegalovirus (CMV) immediate-early promoter. They report protective levels of anti-HBs at birth, and for at least 4 months thereafter [33], further suggesting that TPGD has promise in the prevention of pertussis, hepatitis, and various other infections that occur in infants and animals. This strategy may also be effective for generating disease resistance in farm animals. 

### 2.4. Gene Delivery Cargo and TPGD 

Representative candidate in vivo gene delivery systems for specific nuclease-based genome editing in TPGD are shown in Table 2. Almost all of these systems have been applied in genome editing experiments [7,34,35,36,37,38,39,40,41,42,43,44,45,46,47,48,49,50,51,52]. 

In vivo gene delivery can be performed using non-viral [53] or viral approaches [54], which depend on the use of plasmids or adenovirus, AAV, retrovirus, and lentivirus vectors, respectively [55]. To deliver exogenous plasmid DNA into a fetus via the placenta, cationic lipid transfection reagents are often used, because the placenta provides selective exchange of soluble blood-borne substances through the apposition of uterine and trophoblastic vascularized parts. Some lipids are known to cross the placenta via pinocytosis [56]. Accordingly, Tsukamoto et al. (1995) used Transfectam reagent (IBF Biotechnics Inc., Savage, MD, USA), which is a cationic lipopolyamine containing dioctadecylamidoglycylspermine [18]. Kikuchi et al. (2002) and Nakamura et al. (2019) used FuGENE 6 reagent (Promega KK, Madison, WI, USA), which is a proprietary blend of lipids and other components with very low cytotoxicity that permits high levels of gene expression without adversely affecting cell viability [19,21]. Cornford et al. (2016) reported TPGD-based plasmid DNA delivery and targeting to fetal brains using lipid reagents. Specifically, they covered the surfaces of Trojan horse liposomes (THL) with several thousand strands of polyethylene glycol conjugated to a monoclonal antibody against a brain-specific receptor [28]. Their modified THL were stable in the blood and successfully delivered the gene of interest (GOI) to fetal brains by binding to the target fetal brain receptor. 

Low efficiency of transduction is the main problem with in vivo non-viral gene transfer systems. To overcome this problem, Picconi et al. (2014) first employed recombinant (r) AAV, in which the GOI is expressed under the control of a kidney-specific promoter [26]. To achieve TPGD, they administered rAAV-GOI vector to dams by tail-vein injections and then observed transgene expression in fetal kidneys. In these experiments, rAAV-GOI vector expression was nearly 12-fold increased over controls, and the GOI was not significantly expressed in any other tissues. In addition, the GOI was stably expressed until 12 weeks of age. Thus, maternal tail-vein injections of rAAV may represent a possible avenue for the delivery of gene therapy vectors, because it readily crosses the placental interface and produces stable expression. 

In a study by Srivastava et al. (2004), T7 phages crossed the placental barrier to fetal tissues at E14 and were detectable within 30 min of TPGD [22]. T7 phages were observed in fetal liver, heart, brain, gut, and lung tissues, with high titers in heart, and liver tissues, and the lowest titer in the brain. Because liver and heart tissues are functional in E14 fetuses, greater uptake of T7 phages by these tissues may reflect increased blood flow. This finding suggests that TPGD may be viable at E14, when introductions of plasmid DNA were relatively ineffective [21]. Moreover, T7 phages were rapidly cleared in dams [57], suggesting limited induction of maternal immunity. 

Among strategies for gene therapy, genome editing-based approaches are increasingly considered [2,7,58]. In vivo genome editing using viral vectors has been performed by several laboratories [59], but these vectors are associated with complications, such as mutagenesis, risk of carcinogenesis, and immunogenicity, as indicated by clinical trials [60,61]. As an alternative, AAV is generally considered a safe and effective delivery vehicle and was the vector of choice for >100 clinical trials [62]. This vector does, however, limit the cloning capacity (~4.7 kb) of the vector [63]. For the CRISPR/Cas9 system, the packaging capacity of AAV is limited because the largest component of the CRISPR system, the *Streptococcus pyogenes* Cas9 (*SpCas9*) nuclease gene that is a widely used gene, has a size of 4.2-kb [64]. To overcome this size limitation, many laboratories have explored new types of nucleases (smaller SpCas9 orthologues) and used them in AAV-based CRISPR systems (hereinafter referred to as “AAV-CRISPR”) [65]. For example, *Staphylococcus aureus* Cas9 (*SaCas9*) gene, which is 1 kb shorter (total 3.16 kb) than *SpCas9*, is the most widely used gene and has genome editing ability similar to that of canonical *SpCas9* [48]. In addition, the *Campylobacter jejuni* Cas9 gene (*CjCas9*; 2.95 kb) [66] and *Neisseria meningitides* Cas9 gene (*NmeCas9*; 3.6 kb) [45] are also well known. Therefore, due to safety, simplicity, and flexibility, non-viral delivery systems and AAV-CRISPR are considered promising alternatives, although the development of an efficient in vivo cargo that can deliver these genome editing elements to targeted cells remains a challenge for future studies.

In conclusion, many cases show successful TPGD using plasmid DNA complexed with commercially available cationic lipid reagents. Furthermore, AAV-CRISPR may be a promising tool for in vivo genome editing because it is possible to use a small *Cas9* gene in this system.

## 3. Mechanism of TPGD 

During TPGD, the placenta plays an important role in the transport of nucleic acids from the blood stream of pregnant dams to post-implantation embryos. The placenta is a highly specialized tissue that contributes to fetal developing by controlling the flow of nutrients through the umbilical cord to the uterine wall, by eliminating metabolic decomposition products and by mediating exchange gas between maternal and fetal circulatory systems [67]. 

**Figure 1:**
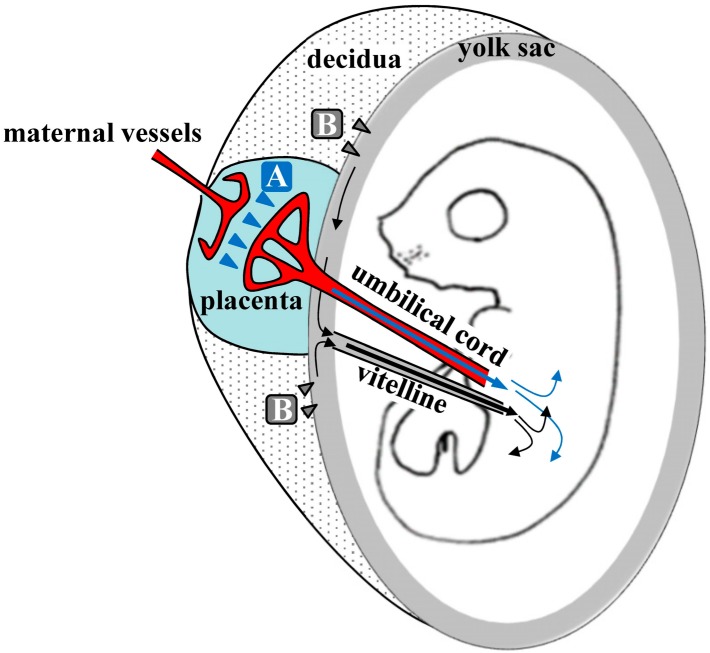
Hypothetical mechanism of transplacental gene delivery (TPGD) as suggested by Kikuchi et al. [21]; Following TPGD on E12.5, when placental circulation is established, intravenously injected plasmid DNA/lipid complexes may be transferred from maternal blood to the fetus via at least two routes. Flow via the placenta to the embryo is indicated by the blue arrowheads (area A); injected plasmid DNA is transferred beyond the blood-placenta barrier (BPB) and enters the umbilical cord. Flow from the decidua to the yolk sac is indicated by the gray arrowheads (area B); some DNA becomes trapped in yolk sac and is transferred to the embryo after the establishment of functional placental circulation.

Kikuchi et al. (2002) speculated about the mechanisms underlying TPGD [21], as shown schematically in Figure 1. They suggest that on E5.5 to E9.5, plasmid DNA is introduced with poor efficiency because the placenta is immature. At this time, nutrients, including lipids, are known to be taken up by the visceral endoderm (VE) or yolk sac, and are then transported to embryos by diffusion or vitelline circulation [68,69]. DNA/lipid complexes in maternal blood may also be transferred to embryos via the VE or yolk sac (arrowheads in Figure 1). However, under these conditions, most DNA/lipid complexes may be trapped in the VE, and small amounts may be taken up and transported to the embryo by vitelline circulation. In contrast, transfer of nutrients commences at the placenta during E10.5 to E13.5. As shown by arrowheads in area A of Figure 1, some plasmid DNA/lipid complexes may be transferred to the umbilical vein at the placenta and then into embryonic circulation. Yet some plasmid DNA/lipid complexes may also enter the blood vessels of the decidua (arrowheads in area B in Figure 1). Tail-vein injections of trypan blue dye demonstrated that some exogenous DNA can be transferred to the embryo via the vitelline veins, but most is trapped at the yolk sac, especially at the portion that is proximal to the placenta. Hence, establishment of placental circulation may account for increased transfer of plasmid DNA/lipid complexes on E12.5 and thereafter. Kikuchi et al. (2002) also suggest that yolk sac circulation may function as a route for the transfer of DNA/lipid complexes from maternal circulation to the fetus [21]. 

**Figure 2:**
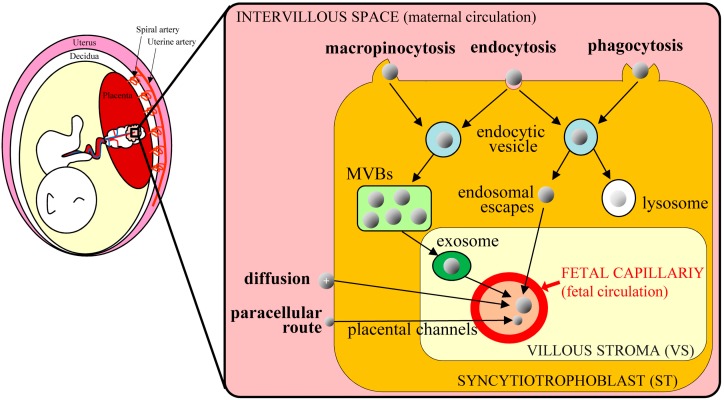
Schematic of nanoparticle transplacental transport mechanisms in humans (based on Zhang et al. [71]); nanoparticles in the maternal circulation cross the placental barrier and are transported to the fetus via various routes. The transcellular route is mediated by endocytosis and exocytosis. Nanoparticles are taken up via macropinocytosis, endocytosis, and phagocytosis in syncytiotrophoblast (ST) cells and are then exocytosed from endocytic vesicles through the multivesicular bodies (MVBs)-related secretion and endosomal escape. After entering the villous stroma (VS), nanoparticles cross endothelial cells of fetal capillaries by diffusion or via exosomes and enter the fetal blood. Some cationic nanoparticles can move toward fetal capillaries by simple diffusion, reflecting electrostatic interactions with cell membranes. Very small nanoparticles can pass ST cells through placental channels and enter the VS through the paracellular route.

Because the placenta is the most species-specific organ, hypotheses that are generated from rodent experiments extrapolate poorly to humans [70]. However, accumulating evidence suggests that nanoparticles in the maternal blood stream can be transferred to the fetus via the placenta. Transfer of nanoparticles through the placental barrier requires passage through syncytiotrophoblast (ST) and villous stroma (VS) layers, and through endothelial cells of fetal capillaries, evoking paracellular and transcellular pathways, respectively [71]. Nanoparticles of less than 25 nm in diameter can pass through the paracellular pathway and penetrate ST layers via passive diffusion, thus entering the VS layers with ease. Cationic nanoparticles are also increasingly considered as vehicles for gene delivery and may directly fuse to ST layers, because basal ST membranes are negatively charged. Other nanoparticles usually employ the transcellular pathway, as exemplified by endocytosis and exocytosis. Alternatively, nanoparticles may be taken up by STs via phagocytosis, clathrin-mediated endocytosis, caveolae-mediated endocytosis, or micropinocytosis. However, these routes are subject to endosomal escape pathways, lysosomal secretion, and multivesicular body (MVBs)-related secretions. In all cases, nanoparticles diffuse into fetal circulation through endothelial cells of fetal capillaries (Figure 2). 

In human placenta tissues, most drugs of less than 600 Da are known to reach the fetus by passive diffusion, whereas drug molecules of over 1000 Da [72] require transport via specific receptor-mediated pathways on ST surfaces. Macromolecules, such as immunoglobulin G antibody (~150 kDa) and vitamin B12 (1.3 kDa), are known to cross the placenta [73]. Hence, such receptor-mediated transfer systems may be critical for delivery of large molecules using TPGD in humans. The mechanisms underlining transplacental transport of substances remain poorly understood, however, and further research will be needed prior to application of TPGD in humans.

## 4. Present Status of TPGD

### 4.1. CRISPR/Cas9 System

Among currently available genome editing tools, CRISPR/Cas9 is widely used to manipulate GOI in a variety of cells and organisms [74,75,76,77,78,79]. It requires the use of synthetic gRNAs that bind to specific chromosomal DNA sites with Cas9 endonuclease [80,81]. The desired target sequence is recognized by the gRNA/Cas9 complex and must immediately precede a 5’-NGG protospacer adjacent motif (PAM) [82]. To date, both components (gRNA and Cas9) have been delivered to cells as single plasmid carrying gRNA sequence and Cas9 gene, or as gRNA and Cas9 mRNA or protein (Cas9 alone). Beside the plasmid for delivery of CRISPR/Cas9 components, AAV-CRISPR is frequently used for this purpose [65]. Interestingly, Yin et al. developed an all-in-one AAV-based vector (carrying four gRNA-expressing cassettes together with a SaCas9-expressing cassette) that can potentially contribute in editing multiple target genes [83]. After the incorporation of these CRISPR/Cas9 components into the cell, gRNA and Cas9 form ribonucleoprotein (RNP) complexes that introduce double-stranded breaks (DSBs) at target sites of the host chromosome. These DSBs are then repaired by nonhomologous end-joining (NHEJ) [84] or gene addition or repair by homologous recombination using an exogenously supplied repair template [85]. 

### 4.2. TPGD-GEF

**Figure 3:**
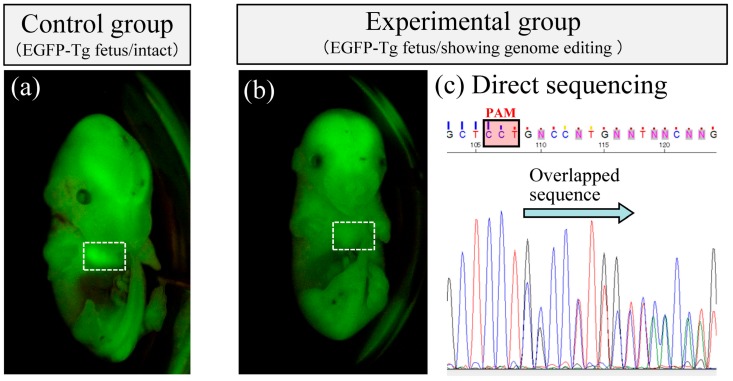
Successful genome editing in fetal cardiac cells after TPGD for acquiring genome-edited fetuses (TPGD-GEF); DNA/lipid complex solutions containing plasmids encoding Cas9 and gRNA targeted to enhanced green fluorescent protein (*eGFP*) cDNA were injected into tail-veins of pregnant dams (at E12.5) containing EGFP transgenic fetuses. The white dashed boxes in (a) and (b) indicate heart. The heart exhibited strong fluorescence in wild-type (intact) fetuses (**a**), whereas fluorescence was greatly reduced in some fetuses of experimental group (**b**). Sequence analyses of PCR products (corresponding to the 5’ region of the *eGFP* sequence) from fetuses (**b**) revealed overlapping electrophoretograms (indicated by arrows) immediately upstream of the protospacer adjacent motif (PAM; **c**). These results indicate the presence of genome-edited and unedited sequences in fetuses with reduced fluorescence in heart tissues.

In almost all experiments using TPGD, plasmid DNA is not incorporated into the genome and its expression in fetuses is transient [18,26]. This property is advantageous for CRISPR/Cas9-based genome editing, which requires transient incorporation into cells, but does not require chromosomal integration of its components. Using TPGD and all-in-one plasmid DNA carrying elements of the CRISPR/Cas9 system, Nakamura et al. (2019) were the first to induce CRISPR/Cas9-mediated mutations in target loci of fetal cardiac cells [19]. This was achieved after tail-vein injections of solution containing single plasmids (pCGSap1-eGFP) complexed with FuGENE6 into pregnant mice on E12.5 [19]. The plasmid construct pCGSap1-eGFP can express Cas9 and gRNA that targets *eGFP* cDNA. All fetuses express EGFP systemically, because they are heterozygous (Tg/+) for the transgene. Thus, the delivery of the CRISPR system targeted to the *eGFP* in fetuses reduces the expression of EGFP due to genome editing of the *eGFP* genomic sequence. In the study by Nakamura et al., twenty-four fetuses were isolated from three pregnant females at 2 days after gene delivery, and three of these were found to have reduced fluorescence in their hearts (Figure 3). Genotyping of these hearts revealed the presence of the transgene construct (*Cas9* gene) in all samples. Furthermore, all three samples exhibited mutations at the target loci, although normal cells were also present. This novel approach requires further improvement as described in Section 5.2, for instance, but may ultimately offer a useful tool for developing animal models of heart disorders and for fetal gene therapy for congenital heart defects such as hypertrophic cardiomyopathy (HCM).

## 5. Application of TPGD-GEF to Manipulations of Fetal Cells 

### 5.1. Fetal Gene Therapy 

Advances in prenatal diagnoses have led to the identification of patients with increased risks of chromosomal anomalies and other genetic diseases. Encouragingly, prenatal gene therapy (fetal gene therapy) could overcome some of the conditions leading to fetal damage. However, applications of fetal gene therapy await resolution of several issues, specifically relating to moral and ethical questions, effects on the reproductive system of the fetus and fetal growth, and concerns about potential abortions. In utero gene delivery, which was conceived in the 1990s, and TPGD (also TPGD-GEF) can be applied during embryonic stages from around E10 in mice, during which fetal structures become evident and several organs become visibly discernible. Indeed, fetal somatic cells transfected with exogenous nucleic acids were easily traceable [86], and in utero gene delivery is currently being assessed in a clinical trial (NCT02453477).

No clinical trials for in utero gene delivery using genome editing technology have yet been performed, although many animal experiments are ongoing. Rossidis et al. was the first to achieve virus-mediated delivery of CRISPR/Cas9 components to mice in utero and demonstrated therapeutic editing of two metabolic genes that cause neonatal death [87]. Their experiments indicate the potential to prenatally treat genetic diseases that result in significant morbidity and mortality before or shortly after birth. Alapati et al. similarly demonstrated that the CRISPR/Cas9 system can be used to perform gene editing during tissue development following in utero intra-amniotic delivery of CRISPR/Cas9 reagents that rescued animals from perinatal lethal monogenic lung disease [88]. This approach targets the lung, and preferentially edits DNA in pulmonary epithelial cells and secretory airway epithelial cells. These investigators showed that in utero gene editing can ameliorate congenital lung disease, with improved survival rates in model mice. These proof-of-concept studies demonstrate the potential of this new approach for the treatment of congenital genetic disorders.

TPGD-GEF, which enables noninvasive induction of genome editing (or gene correction) in fetuses, can be potentially applied to fetuses with congenital disorders. As mentioned previously, TPGD-GEF was effective for CRISPR/Cas9-mediated mutations at a target locus (*eGFP* genomic sequence) in embryonic cardiac cells, but it produced a mixture of unedited and edited cells. Perhaps the functions of embryonic cardiac cells can be manipulated by enhancing or weakening the expression of endogenous target genes using TPGD-GEF. For example, HCM was prevented in mice with only 25% reductions in mutant transcript levels of the myosin heavy chain (MHC) [89]. HCM is caused by dominant point mutations in the MHC gene and is considered a leading cause of sudden unexpected non-violent death. TPGD-GEF may offer an alternative to fetal gene therapy for HCM.

### 5.2. Improvements of TPGD Efficiency

**Figure 4:**
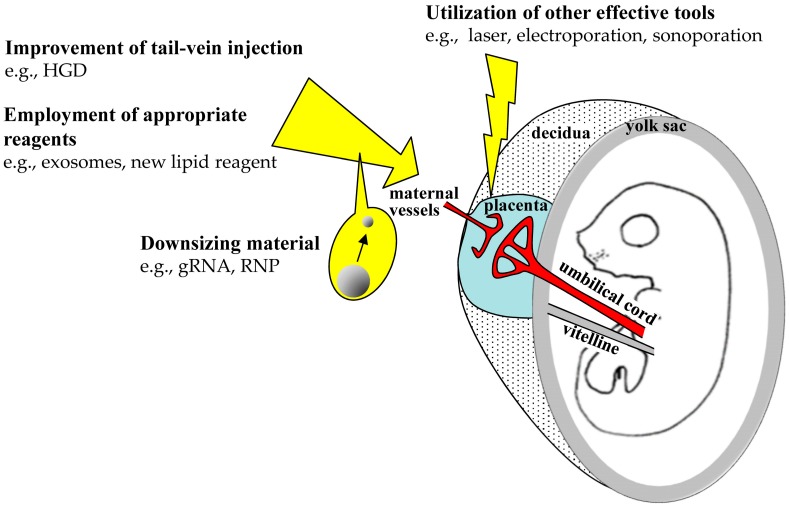
Schematic of possible improvements of TPGD; downsizing of materials, improvements of injection procedures, employment of reagents that are suitable for in vivo gene delivery, and utilization of other effective methods are considered.

Low and unstable efficiency is the main shortcoming of TPGD, as suggested by Rui Maeda-Mamiya et al. [23]. Accordingly, Nakamura et al. (2019), transfected fetuses with a plasmid carrying genome editing components using TPGD but with a gene delivery efficiency of only 25% [19]. Hence, for practical use, further improvements are strictly required (Figure 4). To this end, TPGD efficiency may vary between stages of pregnancy and could be improved with reagents that are more suitable for in vivo gene delivery or by administration of increased amounts of DNA. New techniques, such as hydrodynamics-based gene delivery (HGD), may also help to address this issue. HGD employs hydrodynamic pressure induced by volumes and flows of injections to facilitate intracellular gene transfer in vivo [90,91,92,93]. According to Kertschanska et al., diameters of rodent placental pores and channels range from 15 to 25 nm under normal intravascular pressure [94]. HGD enlarges pores of cells [95] and may therefore enlarge placental pores, through which large amounts of nucleic acids from maternal blood could be transferred to fetuses. To this end, Efremov et al. (2010) employed HGD for TPGD and found that expression levels of a reporter gene increased by 25% in the fetus [24]. In our preliminary experiments, we performed HGD-based TPGD in pregnant mice (at E12.5) and observed increased rates of genome-edited fetuses but concomitant increases in maternal fatalities (unpublished).

Decreasing the sizes of nucleic acid-containing particles may improve the efficacy of TPGD. For example, exosomes of 30–100 nm are frequently used as vehicles for in vivo and in vitro delivery of nucleic acids [96,97]. In the blood stream, exosomes are known to be transferred to brain tissues via the blood–brain barrier (BBB), which functions as a blood–tissue barrier that is similar to the blood–placental barrier (BPB) [98]. Thus, nucleic acid delivering exosomes introduced in the blood streams of pregnant dams will likely be transferred to the fetus with ease via the transplacental barrier. In addition, laser radiation of the brain was shown to enhance the transfer of substances into the brain [99], suggesting that it opens the BBB. Accordingly, laser irradiation of pregnant dams may enhance transplacental transport of nucleic acids from the maternal blood stream to the fetus. TPDG may also be improved in Tg mice expressing Cas9 systemically. Such Tg animals have been produced in ours [100] and other laboratories [101,102]. Moreover, in vivo genome editing in these mice was possible following tissue delivery of gRNA alone [103]. Because gRNA molecules are small, TPGD using gRNA could lead to increased rates of genome editing in fetuses. 

## 6. Concluding Remarks

In this review, we summarize the development of TPGD and describe its utility for CRISPR/Cas9-based genome engineering of fetal cells. Genome engineering can be applied in experimental small animals and in domestic animals and has the potential to be used as a human fetal gene therapy. In future, it may be possible to manipulate, (i) fetal genomes of domestic animals, which are more difficult to manipulate than fetuses from small animals such a mice, and (ii) genomes of fetuses from animals for which in vitro cultivation systems are not fully established. In the latter case, TPGD could be used as a noninvasive method for transplacental gene transfer to fetuses and may be a promising technique for fetal gene therapy. Notably, TPGD using plasmids encoding viral antigens strongly induces protective immunity, potentially preventing maternal–fetal transmission. 

Shortcomings of TPGD include the low efficiency of gene delivery to fetuses. Among solutions to this problem, reductions in molecular sizes of cargos will help to avoid trapping of nucleic acids at the BPB. Recently, RNP-based delivery of CRISPR/Cas9 was applied with reduced toxicity and high efficiency of gene engineering at the target locus, as compared to the other delivery systems [104]. TPGD with biodegradable new materials incorporating RNP, such as exosomes, DNA Nanoclews [105], and some chemical delivery particles [106,107], may also facilitate fetal gene therapy. Furthermore, TPGD using rAAV with RNP is promising because several types of rAAV vectors have been developed [65,108]. For example, Yin et al. constructed an all-in-one AAV-based vector (carrying four gRNA-expressing cassettes and a SaCas9-expressing cassette), which allows editing of multiple target genes at once in a fetus [83]. On the other hand, TPGD is the risk of maternal transduction [109]. To address this, tissue-specific promoters could be used to drive the expression of GOI [26,91]. Alternatively, plasmid DNA delivery systems that employ receptor-mediated endocytosis of DNA complexes with cationic peptide conjugates [110] may limit maternal transduction, while achieving targeted gene transfer to fetuses. As mentioned above, TPGD and TPGD-GEF are still in the process of improvement but have a great potential as future fetal gene therapies.

## Figures and Tables

**Table 1:** Summary of transplacental gene delivery (TPGD)-related experiments.

Pregnant Mice	Injected Time (E) ^1^	Injected Material	GOI ^2^	Reagents Used for Gene Delivery	Note	Year, Reference
ICR	3.0–15.0	Plasmid DNA	Carrying *CAT or lacZ* gene	Commercially available lipopolyamine reagent (Transfectam)	This is the first report concerning TPGD. E9.5 is the day allowing to achieve most efficient TPGD efficiency.	1995 [18]
ICR	11.5	Plasmid DNA	Carrying *lacZ* gene	Commercially available lipopolyamine reagent (DMRIE-C)	Although the transferred efficiency of DNA into embryos were low, expression of the reporter gene was observed.	1999 [20]
BALB/c	5.5, 9.5, 14.5	Plasmid DNA	Carrying gene encoding antigen from HIV-1 or influenza virus	Cationic liposome prepared in-house	DNA-vaccinated mothers confer the antigen-specific immunity to their progeny.	2001 [9]
B6C3F1 ^3^	4.5–13.5	Plasmid DNA	Carrying *Cre* gene	Commercially available lipid reagents (FuGENE6/Lipofectin/DOSPER)	This is the first report that the TPGD can mediate Cre/*loxP*-based recombination even in a fetus.	2002 [21]
BALB/c	14	T7 phage particles	none	none	T7 Phage were detected in various fetal tissues.	2004 [22]
Multiple strains of mice	6.5	Plasmid DNA	Carrying *DsRed* cDNA and shRNA for *geminin* gene	none	This is the first report that the TPGD is useful for RNAi-based gene silencing in a fetus.	2006 [10]
C57BL/6	8	Plasmid DNA	Carrying *GFP* cDNA	Tetra (piperazino) fullerene epoxide (TPFE)	Injected plasmid DNA was detected in the fetus, but the transfection efficiency was very low.	2010 [23]
C57BL/6	17–19	Plasmid DNA	Carrying *luciferase* gene	Nuclear location signal (NLS)-alarelin peptide	This is the first report that the TPGD coupled with hydrodynamics-based gene delivery (HGD) is useful for efficient transfection of a fetus.	2010 [24]
ICR	5.5–10.5	Plasmid DNA	Carrying *GFP* cDNA and shRNA for *Sry* gene	Polyethylenimines	This report employs HGD and shows that the transfection efficiency is associated with the injection-time, -speed, and -volume.	2012 [25]
C57BL/6	12.5	Recombinant adeno-associated virus	Carrying *GFP* cDNA	None	Kidney-specific GOI expression was observed in a fetus, although the expression was also found in the dam.	2014 [26]
CD-1	8	Adenovirus	Carrying *sFlt-1* gene	None	The authors created disease animal model by TPGD to evaluate the role of drugs in preventing the disease.	2014 [27]
C57BL/6	17	Plasmid DNA	Carrying *luciferase* gene or *lac Z* gene	PEGylated immunoliposomes within immunoliposomes bearing 8D3 monoclonal antibodies	Receptor-mediated transport of GOI via placental barrier is possible.	2016 [28]
B6C3F1 ^4^	12.5	Plasmid DNA	Carrying humanized *Cas9* gene and gRNA to *eGFP*	Commercially available lipid reagent (FuGENE6)	This is the first report that the TPGD is useful for inducing genome editing in fetal cardiac cells.	2019 [19]

^1^ The day on which a copulation plug is found is defined as embryonic day 0 (E0). ^2^ Gene of interest; abbreviations: *Cas9*, clustered regularly interspaced short palindromic repeats-associated protein-9 nuclease; *CAT*, chloramphenicol acetyltransferase; *DsRed*, *Discosoma* sp. red fluorescent protein; (*e*)*GFP*, (enhanced) green fluorescent protein; gRNA, guide RNA; HIV, human immunodeficiency virus; *lacZ*, β-galactosidase; *sFlt-1*, soluble fms-like tyrosine kinase-1; shRNA, short hairpin RNA; *Sry*, Sex-determining region Y. ^3^ In this case, female B6C3F1 (a hybrid between C57BL/6 and C3H/He) mice were mated with transgenic males carrying the CETZ-17 transgene (containing *loxP*-flanked sequence). A percentage of fetuses in carried the CETZ-17 transgene. ^4^ In this case, female B6C3F1 (a hybrid between C57BL/6 and C3H/He) mice were mated with transgenic males carrying the CAG-EGFP transgene (chicken β-actin-based promoter (CAG) + *eGFP* cDNA + poly(A) site) in a homozygous (Tg/Tg) state. All fetuses expressed EGFP systemically, because they are heterozygous (Tg/+) for the transgene.

**Table 2:** Representative in vivo gene delivery cargos that are suitable for TPGD-based genome editing.

Delivery System		Representative Advantage	Representative Disadvantage	e.g.,	Application Examples in TPGD	Application Examples in Genome Editing Systems
**Non-viral method**	Cationic lipid	Low cost; great stability; simple and easy handling	Low efficiency; delayed onset	commercially available reagent for gene delivery (FuGENE6, *etc*.)	4 cases reported [18,19,20,21]	Many cases reported [7,34,35,36]
				Immunoliposome	1 case reported [9]	none
				PEGylation	1 case reported [28]	Some cases reported [37,38]
	Chemical reagent	Easy to produce; large packaging capacity	Low targeting efficiency; toxic	Carbon nanotube	1 case reported [23]	none
				Polyethylenimines	1 case reported [25]	Some cases reported [39,40]
	Polymers	easy to optimize	Cannot be applied to deliver the native form of Cas9 protein	Peptide	1 case reported [24]	Many cases reported [7,35,41,42]
	Secretion	High efficiency; tissue-specificity	There are many unexplained parts	Exosome	None	Some cases reported [43,44]
Viral method	Virus	Generally considered a safe and effective delivery vehicle	Low packaging capacity (less than 4.7 kb); difficulty in production of high-affinity virus targeted to liver	Adeno-associated virus	1 case reported [26]	Many cases reported [45,46,47,48]
		High efficiency; high packaging capacity	High immunogenicity	Adenovirus	1 case reported [27]	Some cases reported [49,50]
		High efficiency	Does not efficiently infect human cells	Bacteriophage	1 case reported [22]	Some cases reported [51,52]

## Abbreviations

AAV	Adeno-associated virus
BBB	Blood-brain barrier
BPB	Blood-placental barrier
B6C3F1	Mouse hybrid between C57BL/6 and C3H/H
Cas9	Clustered regularly interspaced short palindromic repeats (CRISPR)-associated protein-9 nuclease
CAT	Chloramphenicol acetyltransferase
CMV	Cytomegalovirus
CRISPR	Clustered regularly interspaced short palindromic repeats
DSBs	Double-stranded breaks
DsRed	*Discosoma* sp. red fluorescent protein
EGFP	Enhanced green fluorescent protein
GFP	Green fluorescent protein
GOI	Gene of interest
gRNA	Guide RNA
HBs	Hepatitis B surface antigen
HBV	Hepatitis B virus
HCM	Hypertrophic cardiomyopathy
HGD	Hydrodynamics-based gene delivery
HIV	Human immunodeficiency virus
lacZ	β-Galactosidase
MHC	Myosin heavy chain
MVBs	Multivesicular bodies
NHEJ	Nonhomologous end-joining
NLS	Nuclear location signal
PAM	Protospacer adjacent motif
rAAV	Recombinant adeno-associated virus
RNAi	RNA interference
RNP	Ribonucleoprotein
sFlt-1	Soluble fms-like tyrosine kinase-1
shRNA	Short hairpin RNA
Sry	Sex-determining region Y
ST	Syncytiotrophoblast
TALEN	Transcription activator-like effector nucleases
Tg mouse	Transgenic mouse
THL	Trojan horse liposome
TPFE	Tetra (piperazino) fullerene epoxide
TPGD	Transplacental gene delivery
TPGD-GEF	Transplacental gene delivery (TPGD) for acquiring genome-edited fetuses
VE	Visceral endoderm
VS	Villous stroma
ZFN	Zinc-finger nuclease
